# Circadian Clocks and the Interaction between Stress Axis and Adipose Function

**DOI:** 10.1155/2015/693204

**Published:** 2015-04-27

**Authors:** Isa Kolbe, Rebecca Dumbell, Henrik Oster

**Affiliations:** Chronophysiology Group, Medical Department I, University of Lübeck, 23538 Lübeck, Germany

## Abstract

Many physiological processes and most endocrine functions show fluctuations over the course of the day. These so-called circadian rhythms are governed by an endogenous network of cellular clocks and serve as an adaptation to daily and, thus, predictable changes in the organism's environment. Circadian clocks have been described in several tissues of the stress axis and in adipose cells where they regulate the rhythmic and stimulated release of stress hormones, such as glucocorticoids, and various adipokine factors. Recent work suggests that both adipose and stress axis clock systems reciprocally influence each other and adrenal-adipose rhythms may be key players in the development and therapy of metabolic disorders. In this review, we summarize our current understanding of adrenal and adipose tissue rhythms and clocks and how they might interact to regulate energy homoeostasis and stress responses under physiological conditions. Potential chronotherapeutic strategies for the treatment of metabolic and stress disorders are discussed.

## 1. Introduction

In order to optimise survival in a temporally variable environment, many behavioural and physiological processes have evolved to have an optimally timed expression. These rhythmic processes may oscillate over the course of a year, a month, or a day and synchronise to the external environment in order to save energy at times when these processes would be inappropriate. Circadian clocks (from Latin* circa diem*, about a day) influence almost all biological processes from sleep-wake rhythms, food intake, energy metabolism, body temperature, immune function, and cardiovascular function to cell proliferation [1] and allow for accurate coordination of these processes with a period of approximately 24 h in the absence of external timing signals. Synchronisation of the circadian clock to the external environment occurs via stimuli such as light and food intake, the so-called zeitgebers (from German, time giver). It is currently accepted that the circadian regulation of physiology and behaviour occurs in a hierarchical manner, with a master clock residing in the paired structure of the suprachiasmatic nuclei (SCN) in the anterior hypothalamus, which receives light input via the retinohypothalamic tract, and downstream subordinate clocks that occur in various tissues. In mammals at least, it is considered that most tissues and cells contain functional molecular clockwork similar to that of the SCN [2], and within the hierarchical organisation of circadian timekeeping the adrenal gland plays a key role, since adrenocortical glucocorticoid rhythms are thought to synchronise clocks in various peripheral and central tissues [3–5].

According to the current model, the molecular circadian clock consists of interlocked transcriptional-translational feedback loops (TTLs), with the positive arm being composed of the transcription factors circadian locomotor output cycles kaput (CLOCK) or neuronal PAS domain-containing protein 2 (NPAS2) and brain and muscle aryl hydrocarbon receptor nuclear translocator-like 1 (BMAL1; also called ARNTL or MOP3). These form heterodimers via PAS domains to activate transcription of genes containing circadian transcription factor binding* E-box* elements including* Period* (*Per 1–3*) and* Cryptochrome* (*Cry1/2*), expressed in subjective daytime and comprising the negative arm of the core TTL. PER/CRY complexes translocate to the nucleus where they accumulate over time and inhibit CLOCK-BMAL1 or NPAS2-BMAL1 activity. In this way* Per* and* Cry *transcription is suppressed during subjective night-time, and the cycle completes as* Per/Cry* complexes are degraded towards the morning when CLOCK/NPAS-BMAL1 inhibition is released. Further, stabilizing TTLs include the nuclear hormone receptors REV–ERB*α* and REV–ERB*β* and ROR*α* which regulate* Bmal1* expression and the basic helix-loop-helix transcription factors DEC1 and DEC2 [6, 7].

The hypothalamic-pituitary-adrenal (HPA) axis and glucocorticoids in particular have wide ranging effects on physiology and behaviour. Glucocorticoids are involved in the stress response, being secreted rapidly along with epinephrine under acute stress, and exert influence on metabolic functions such as glucose homeostasis, as well as immune and cognitive processes [8–11]. Therefore, the disruption of glucocorticoid rhythmicity is implicated in several pathologies. An interaction between the HPA axis and adipose physiology has long been proposed, and the effect of adipokines, adipose derived cytokines, on physiology, particularly with regard to metabolic disorders, is an area of active research.

In modern industrial societies, life-style and work demands increasingly interfere with endogenously determined circadian rhythms. Off-shift workers suffer from disrupted sleep-wake and eating rhythms. Frequent intertime zone travel leads to a misalignment between internal and external time. Moreover, work- or leisure-associated early or late wake times have led to the wide-spread phenomenon of social jet lag, where internal rhythms are overrun by artificial zeitgebers resulting in accumulation of sleep debt, for example, during the working week. Together this has led to an increased interest in the interaction of circadian rhythms and health parameters. Several metabolic disorders are associated with circadian disruption [12–14], and obesity in particular is often accompanied by altered HPA axis rhythmicity [15–18]. This review will discuss the circadian aspects of HPA axis regulation and adipokine secretion, their known interactions, and the potential consequences for human physiology.

## 2. Regulation of the HPA Axis Circadian Rhythm

Glucocorticoids are key components of the hypothalamic-pituitary-adrenal (HPA) axis and act as the final effectors of this axis on other systems. The HPA axis is regulated in a classical endocrine negative feedback loop. Briefly, the production of the neuropeptides corticotrophin-releasing hormone (CRH) and arginine vasopressin (AVP) occurs at the paraventricular nucleus of the hypothalamus (PVN) under the circadian influence of the SCN and stress-induced signals from the brainstem and the limbic forebrain [19, 20]. Both reach the pituitary via the hypophyseal blood portal system and stimulate corticotrophin (adrenocorticotropic hormone (ACTH)) secretion into the circulation. ACTH in turn stimulates the production of glucocorticoids at the adrenal cortex, and glucocorticoids provide negative feedback by inhibiting the production of CRH at the PVN and ACTH at the pituitary. Steroidogenesis occurs at the adrenal cortex where ACTH binds to the melanocortin 2 receptor (MC2R) leading via cAMP-PKA signalling pathways to the transcription of steroidogenic genes such as cholesterol side chain cleavage enzyme (*CYP11A1*) and steroidogenic acute regulatory protein (*StAR*). In turn the key rate limiting stage of glucocorticoid production occurs with the transportation of cholesterol into adrenocortical cells via scavenger receptor class B member 1 (SR-B1) and low-density lipoprotein (LDL) receptors, and into the mitochondria by StAR.

The rhythmic release of glucocorticoids into the circulation occurs in a robust circadian fashion under nonstressed conditions [21]. Such is the robustness of the circadian cortisol rhythm in humans, that it has been used as a marker for the general circadian health of an individual [22–24]. Circadian glucocorticoid rhythms peak slightly before the onset of the active phase in the late light phase for nocturnal species (e.g., rats and mice) and in the late dark phase for diurnal species including humans [25]. A true circadian rhythm, this glucocorticoid secretion pattern persists in a constant environment and relies on an intact SCN, first demonstrated in rats [26, 27]. The circadian pattern is overlaid by an ultradian rhythm consisting of pulses averaging between 80 and 110 minutes in humans and 50 and 60 minutes in rats. This ultradian rhythm has been demonstrated* in vivo* to be independent of the SCN or any connection between the hypothalamus and pituitary [28, 29] and is controlled by negative feedback whereby glucocorticoids signal at pituitary glucocorticoid receptors (GRs) to suppress ACTH secretion [30]. The GR (also called NR3C1) is expressed throughout the body but is absent in the SCN [3] and mediates the acute effects of glucocorticoids which bind only during ultradian pulse peaks and rapidly dissociate from this receptor [31]. In addition, glucocorticoids also bind to the mineralocorticoid receptor (MR, sometimes called the corticosterone receptor) with high affinity, which has a more specific tissue distribution [32], allowing for a permissive or long-term activation during the peak of circadian glucocorticoid concentration. There is evidence for the involvement of the MR in adipose tissue function, and glucocorticoids in particular are thought to act via this receptor to modulate adipogenesis [33]. In addition to glucocorticoids, the mineralocorticoid aldosterone, important in the regulation of blood pressure, is known to have a circadian rhythm and act via the MR [34, 35]. Mice lacking* Bmal1* are hypotensive [36] and in mice lacking both* Cry1* and* Cry2* the aldosterone rhythm is lost, being constantly high, and under a high salt diet these mice become hypertensive [34].

The circadian regulation of glucocorticoid release is not only controlled by the HPA axis but is under the influence of several factors. Aside from influencing the hypothalamic component of the HPA axis, the SCN also exerts its effects on the adrenal via the autonomic nervous system (ANS), and furthermore, the adrenal gland is in possession of its own circadian clock. Within the HPA axis, circadian rhythms exist in the concentration of circulating ACTH [37] and CRH expression in the PVN [38], but these rhythms do not synchronise well enough to explain the regulation of rhythmic glucocorticoid concentration [38, 39]. Moreover, glucocorticoid rhythms persist in the absence of rhythmic ACTH [40] or CRH [41]. The importance of ACTH for circadian glucocorticoid secretion should not be completely discounted, however, since ACTH can cause a phase dependent phase delay in adrenal glucocorticoid rhythms* in vitro*, indicated in tissue from* mPER::Luciferase* knockin mice [42], and ACTH can directly stimulate* BMAL1* and* PER1* expression in human explant adrenals [43]. In their 2014 study, Yoder and colleagues were able to stimulate a phase delay only, and so any effect of ACTH is independent of entrainment to the light cycle. Furthermore, the phase delay only occurred when stimulated at circadian time (CT) 18 in their experiments, and so although unlikely to be the main entrainment factor, ACTH may play a role in resetting the adrenal rhythm under certain conditions; that is, a stress response occurring at this vulnerable time may stimulate a phase delay of adrenal clocks.

The SCN exerts autonomic control over glucocorticoid rhythms via preautonomic PVN neurons projecting to sympathetic preganglionic intermediolateral neurons of the spinal cord and splanchnic nerve innervation of the adrenal [44]. The importance of the autonomic influence on glucocorticoid rhythms has been demonstrated in hypophysectomised rats [45, 46] and by splanchnic nerve transections [47, 48]. The mechanism of ANS stimulation of glucocorticoid rhythms remains to be elucidated and is reviewed in greater detail elsewhere [49].

Preceding the description of the molecular circadian clock, a robust circadian rhythm of steroid secretion was first demonstrated in isolated adrenal glands of the Syrian hamster [50], and more recently, rhythmic expression of clock genes has been well demonstrated in the adrenal cortex of both rodents and primates [51–56]. Approximately, 10% of the murine adrenal transcriptome shows circadian variation, including genes involved in cholesterol transport, steroidogenesis, and ACTH signalling [54]. Mice lacking genes from the negative arm of the TTL are hypercortisolic [5, 57] and in contrast, those lacking genes encoding BMAL1 or CLOCK are hypocortisolic [58, 59]. Clock deficient mice lack the rhythmic expression of StAR, being constantly high in* Per1/2* ^−/−^ and* Cry1/2* ^−/−^ mice [60], while* Star* shows reduced expression in* Bmal1* ^−/−^ mice [58]. The relative importance of the central and local adrenal circadian clock in the regulation of glucocorticoid rhythm is an area of active research. The adrenal clock appears to have an important role to play in the regulation of adrenal ACTH sensitivity, being rhythmically regulated by a gating mechanism via the local circadian clock. In consequence, adrenals from clock-deficient mice without functioning* Per2* or* Cry1* have defective corticosterone synthesis when transplanted to wild-type adrenalectomized hosts [61]. The importance of StAR as a link between the molecular clock and steroidogenesis has been demonstrated by the overexpression of* Clock* and* Bmal1* in the mouse adrenocortical Y1 cell line, which led to increased StAR expression and steroid production, and which was then inhibited upon application of antisense StAR oligodeoxynucleotides [60]. In the same study, Son and colleagues were able to demonstrate that an adrenal conditional knockdown of* Bmal1* led to loss of StAR and intra-adrenal corticosterone rhythmicity in the absence of a light cue.* Ex vivo* cultured adrenals from mice lacking* Bmal1* have blunted corticosterone secretion in response to ACTH [58]. Similarly, in explant studies on primate adrenal tissue, the knockdown of* Cry2* and subsequent loss of* Bmal1* expression were accompanied by blunting of ACTH stimulated increases in cortisol secretion as well as StAR and 3ß-hydroxysteroid dehydrogenase protein expression [62].

## 3. Glucocorticoid Synchronisation of Peripheral Clocks

The HPA axis, through glucocorticoids, exerts influence on many important biological processes, and glucocorticoids are proposed to have an important synchronizing role on peripheral circadian rhythms [3, 63]. Glucocorticoids have been found to broadly influence gene expression through GR [64]. When activated, the cytoplasmic GR, previously in an inactive complexed state, undergoes conformational changes and, after dimerization, translocates into the nucleus. In the nucleus GR activates transcription of glucocorticoid target genes by binding to glucocorticoid response elements (*GRE*s) [65]. These* GRE*s regulate expression of several genes including some core clock genes such as* Per1, Per2, Npas2*, and* Rev-erbß* [3, 4, 66, 67]. The capability of glucocorticoids to shift the rhythm of peripheral clocks has been demonstrated* in vitro* and* in vivo* with dexamethasone, a synthetic glucocorticoid analogue, inducing clock gene and clock dependent gene expression in rat fibroblasts. Furthermore, on administration to live mice dexamethasone was able to delay or advance the phase of clock gene expression in the liver, kidney, and heart depending on the time of injection [3]. More recently, evidence for the effect of glucocorticoids to influence circadian rhythms of human adipose tissue has also been provided [68].

In addition,* in vitro* studies indicate that molecular clock components are able to negatively regulate the action of GR to influence gene expression. CLOCK/BMAL1 is able to physically interact with GR to inhibit binding to* GRE* sites in order to suppress glucocorticoid stimulated gene expression in human colon cancer HCT116, and human cervical cancer HeLa cells [69]. Similarly* in vitro* and* in vivo* murine studies demonstrate the importance of* Cry1* and* Cry2* for suppression of glucocorticoid stimulated gene expression since both CRY1 and CRY2 are able to interact with the GR in order to oppose activation of this receptor [5].

## 4. HPA Axis Rhythmicity and Energy Homeostasis

Desynchrony between the central SCN clock and peripheral oscillators can be brought about by inappropriately timed food intake. Under normal conditions in mice, feeding is largely restricted to the dark phase. By restricting food access to the light phase, core clock genes such as* Per1, Per2, Per3, Reverbα*, and* Cry1* and clock output genes such as* Dbp* and* Cyp2a5* are phase shifted by 8–12 h in the liver independent of SCN* Per1/2* expression. In the same study, phase shifts were additionally observed for* Dpb* expression in the kidney, heart, and pancreas, while mice fed exclusively in the night had peripheral rhythms similar to those fed* ad libitum* [70]. These shifted peripheral clock rhythms correlate with changes in glucocorticoid signalling, with daytime feeding in mice causing an additional peak in circulating corticosterone in advance of the peak observed at 12 h following lights on (*zeitgeber* time; ZT 12) for* ad libitum* fed animals, and the phase shift in the liver was absent in mice lacking GR in this organ [71]. Outside the experimental context, timing of food intake can be influenced by social or environmental factors such as shift work, sleep curtailment, or inter-time-zone travel (jetlag). Interestingly, disruption of diurnal feeding rhythms can also be induced by high-fat diet, with mice roughly doubling the proportion of nutrient intake during the light phase after only one week. This alteration of diet is accompanied by dampening of clock gene expression rhythms in liver and fat tissue and altered rhythms of several circulating metabolic factors including corticosterone and leptin [72]. Thus, the timing of food intake, whether caused by environmental factors or influenced by diet composition, is likely important for the maintenance of peripheral and central clock synchrony.

Obesity is associated with a dampened circadian glucocorticoid rhythm in wild type and in genetically obese rodents [73–75] and humans [15–18]. A correlation between the abdominal fat distribution, elevated dietary lipid (and in particular higher saturated fatty acid) content, and disturbance of the HPA axis has been found in women, who have a low variability between morning and evening salivary cortisol. In the same study, women with less difference between morning and evening cortisol samples, as well as preferring food containing more saturated fatty acids, also had higher postprandial cortisol secretion [76].

On the other hand, mice with a genetically disrupted circadian clock display disrupted feeding rhythms and high propensity to metabolic disease [59, 77, 78].* Per1* mutant mice have constantly high corticosterone levels and fail to gain weight as efficiently as wild type animals despite the high body weight-adjusted food intake, suggestive of increased metabolic rate, along with increased glucose metabolism that the authors attribute to the lack of a robust glucocorticoid rhythm [78]. In* Clock* mutant mice, several genes important for appetite regulation lose rhythmic expression in the hypothalamus. This is associated with a strongly attenuated diurnal feeding rhythm as well as increased weight gain on both standard and high fat diet, going along with measurable detrimental effects on circulating metabolic parameters including glucose, cholesterol, triglyceride, and leptin levels [59]. In the* Cry1/Cry2* double-deficient mouse, enhanced vulnerability to diet-induced obesity and metabolic syndrome has been demonstrated. As previously discussed, these mice are hypercortisolic and have impaired glucose metabolism [5]. When kept on a high fat diet, besides gaining more weight, these mice also show increased weight gain relative to food intake, associated with a loss of rhythmicity in metabolic rate, increased fat mass, and insulin secretion [57]. This is in contrast to mice lacking only* Cry1*, however, that are more resistant to diet-induced obesity and show decreased overall fat mass compared to wild type controls [79]. In the same study, Griebel and colleagues found no difference between* Cry2* knockout mice and wild type controls in terms of the response to high fat diet [79]. Interestingly, glucocorticoids may play a role in adipocyte differentiation via the MR, with knockdown of this receptor, but not GR, inhibiting adipogenesis in the murine 3T3-L1 cell line [33].

## 5. Circadian Rhythms in White Adipose Tissue

Quantitatively, most white adipose tissue (WAT) is of either subcutaneous or visceral origin in humans. While subcutaneous depots mainly store energy and provide thermal insulation, internal WAT depots have a higher endocrine activity [80]. White adipocytes are long-term energy stores and accumulate energy in the form of triglycerides (TGs), which are either absorbed directly from the bloodstream or generated within the adipocyte by* de novo* lipogenesis [81]. In the converse process of lipolysis, white adipocytes break down TGs and release the resulting free fatty acids (FFAs) and glycerol into the circulation in order to support other organs during times of energy shortage or acute stress situations [82]. In order to prevent metabolic disorders, lipogenesis and lipolysis in white adipocytes are both tightly regulated since high concentrations of circulating FFAs and TGs can cause lipotoxicity and promote cardiovascular complications, and hyper-uptake of TGs can result in undue increase of adipose tissue mass. Therefore, these processes have evolved to be regulated not solely in response to food intake, but are under control of clock-mediated circadian rhythms [83, 84].

Local adipose transcription rhythms and a number of genes encoding key lipogenesis and lipolysis enzymes have promoters that harbour* E-box* elements, and thus are direct targets of the circadian clock [65]. Of more than 2,000 genomic loci in mouse liver that have been identified to be directly rhythmically regulated by BMAL1 and CLOCK using chromatin immunoprecipitation with parallel DNA sequencing (ChIP-seq), many encode genes which are involved in carbohydrate metabolism (e.g.,* Glut2, Pck1*, and* Gys2*) and lipid metabolism (e.g.,* Dgat2, Lipe*, and* Pnpla2*) [85]. It is still to be determined whether a similar extent of these BMAL1/CLOCK targets can be found in adipose tissue; however, it is known that BMAL1 directly and rhythmically controls the transcription of* Lipe* and* Pnpla2* in WAT. In addition, robust and coordinated expression of circadian oscillator genes (*Npas2, Bmal1, Per1*–*3*, and* Cry1-2*) and clock-controlled downstream genes (*Rev-erbα, Rev-erbβ, Dbp, E4bp4, Stra13*, and* Id2*) has been described for several murine adipose tissues [86]. Furthermore, the molecular clock can be linked to lipid metabolism since PER2 directly and specifically represses the activity of peroxisome proliferator-activated receptor gamma (PPAR*γ*), a nuclear receptor critical for adipogenesis, the regulation of insulin sensitivity, and inflammatory responses. Thus, PER2 is proposed to have an important role to play in the regulation of PPAR*γ*-mediated adipogenesis [87]. The circadian timing system regulates the rate of lipid storage and mobilisation over the course of the day to ensure optimal energy supply and metabolism. The action of adipose tissue is dictated by the active and rest phases of the daily cycle. In humans this means that storage and lipogenesis both occur during the daytime when active food intake allocates energy demands, and reciprocally, the release of glycerol and FFAs is predominantly restricted to the night [84]. In nocturnal mammals, as with most rodents, this process is accordingly reversed [83].

Adipokines are peptide hormones that are produced in and secreted from adult adipocytes, regulating diverse biological processes and underlying the key role of adipose tissue in the regulation of energy homeostasis [88–90]. As with lipid mobilisation, the secretion of numerous adipokines is under circadian control (reviewed in [91]), with leptin and adiponectin being the most prominent adipocyte derived peptide hormones with a distinct diurnal rhythm. These adipokines have diverse actions; they are involved in metabolic regulation and control important aspects of lipid metabolism, glucose disposal, and adipose endocrine function [92–94]. The rhythmicity of adipokine secretion appears to rely on intrinsic circadian oscillators, a combined control via the master pacemaker in the SCN and local control at the level of adipocyte clocks [83].

Leptin can act directly at the hypothalamus, the main region of energy homeostasis regulation in the brain, to increase energy turnover and inhibit appetite [95]. Irrespective of diurnality or nocturnality, for mice, rats, and humans, leptin concentration peaks at night. In humans this is during the normal rest phase, when hunger is suppressed and metabolic turnover in adipose tissue sustains energy supplies. Disrupted leptin secretion is associated with night eating syndrome, an eating disorder where the patient's sleep cycle is disrupted and significant quantities of food are consumed during the night [96, 97]. Altered leptin concentrations can be also observed in shift workers and this is associated with diminished satiety and obesity [98]. A large part of the metabolic activity of adipose tissue relies on the interaction of leptin, adiponectin, and insulin. Adiponectin is considered important for the modulation of cellular glucose uptake, adipocyte insulin sensitivity, and fatty acid break down [99, 100]. While in lean patients the blood content of adiponectin and leptin oscillates inversely, the adiponectin concentration in obese patients is decreased while leptin is elevated [100].

The expression of GR in white adipose tissue also oscillates with a diurnal rhythm. Under unstressed conditions the mRNA expression of* GR* in rat WAT peaks at the transition from the light/inactive to the dark/active period [101] just as in humans the highest concentration of circulating cortisol is reached in the early active phase [102]. Cortisone reductase (also known as 11*β*-hydroxysteroid dehydrogenase type 1; 11*β*HSD-1) is the key enzyme that locally regenerates the inactive form of 5*α*-tetrahydrocortisol (humans) or 11-dehydrocorticosterone (rodents) [103–105]. While its expression in rat hippocampus oscillates in a circadian manner, rhythms of 11*β*HSD-1 in peripheral tissues could not be determined [105]. The expression of 11*β*HSD-1 is locally controlled and is important for amplifying glucocorticoid feedback to the HPA axis, in addition to influencing glucocorticoid action. Under obese conditions hippocampal oscillation is dampened in rats, and with a disturbed HPA axis function the local 11*β*HSD-1 activity and GC action are also altered [104, 105].

## 6. Interactions between the HPA Axis and Adipose Rhythms

As with the majority of peripheral rhythms investigated, the intrinsic adipocyte clock is synchronised by the SCN via neuronal and endocrine pathways. These SCN pathways to the periphery overlap closely with that of the HPA axis and enable potential interactions. Disrupted HPA axis rhythms are associated with the obese state and, in humans, obesity is correlated with increased glucocorticoid production and degradation, resulting in an overall higher cortisol turnover rate [106]. Furthermore, under stressed or hypocortisolaemic conditions, modulation of adipocyte and adipokine circadian rhythm is likely to occur. Indeed,* GRE* regions have been found in the promoter regions of several genes involved in adipocyte function, including triglyceride accumulation [107] and adipogenesis [108]. Moreover, human explant visceral and subcutaneous adipose tissue clock gene expression rhythms can be altered by dexamethasone administration [68]. In light of this, an interaction pathway with the HPA axis to mediate food intake and body weight via the circadian output of adipocytes is postulated [91]. Since chronic stress is correlated with disrupted or dampened rhythmicity in adipose function [109], this may increase the propensity of weight gain and adiposity further, promoting the development of metabolic disorders such as diabetes under long-term stress exposure[110]. In contrast, the stress response is altered in different metabolic states and in particular is hyperresponsive under obese conditions [111].

Leptin is the best studied of all adipokines to date, and although this is an area of active research, the majority of evidence for adipokine interaction with the HPA axis exists for this peptide. In the healthy state, glucocorticoids are generally considered catabolic and are known to strongly affect leptin expression.* In vitro* glucocorticoid application to isolated adipocytes stimulates the synthesis and secretion of leptin [112–115]. In rats, the peripheral infusion of glucocorticoids induces* Ob* (leptin) gene expression in adipose tissue and overall hyperleptinaemia, resulting in decreased food intake and a reduction in body weight [116, 117]. This is also true for humans, with peripheral administration of glucocorticoids increasing leptin secretion. Therefore, it can be suggested that an impaired adrenal function and resulting hypocortisolemia leads to lower leptin expression in humans and rodents [118–124]. Direct hypothalamic leptin application generates a similar effect on food consumption as reviewed by Friedman and Halaas [125], suggesting that peripheral glucocorticoids may drive leptin mediated appetite effects. In contrast, constant (and therefore arrhythmic) central infusion of glucocorticoids has been demonstrated to increase food intake and body weight, with concurrent hyperleptinaemia, hyperinsulinaemia, and hypertriglyceridaemia that occur in obesity, possibly mediated by altered hypothalamic expression of neuropeptide Y and CRH as proposed for rats [117]. Therefore, a central stimulatory effect of glucocorticoids on food intake can be hypothesised and counter-regulated by elevated leptin levels, while central actions of leptin might be inhibited under glucocorticoid influence. It should be noted that the effect of glucocorticoid action on other factors such as insulin secretion might also play a role in mediating HPA axis effects on energy balance. However in patients with Cushing's disease, characterised by pituitary or adrenal adenoma, hypercortisolemia, and disrupted cortisol rhythms, leptin levels are highly independent of adiposity, and removal of the tumour usually results in lowered levels of both cortisol and leptin [126, 127]. In contrast, adrenalectomized rats experience potent effects on food intake and body weight when leptin is administered, and this effect is inhibited by dexamethasone administration [128]. Taken together this suggests that high levels or disrupted blood rhythms of glucocorticoids may contribute to the leptin resistance observed in obesity. Of note, patients with Cushing's disease develop increased visceral adiposity, while abdominal subcutaneous fat depots are depleted. This suggests that the impact of glucocorticoids on different kinds of adipose tissue varies enormously [129].

Interestingly, leptin deficient* ob/ob* mice show an intact circadian glucocorticoid rhythm despite an overall raised circulating concentration [130] in contrast to the* db/db* mouse, which lacks a functional leptin receptor and along with being hypercortisolaemic it also displays disrupted glucocorticoid rhythms [73]. A fasting induced increase in circulating glucocorticoid and ACTH concentration is accompanied by lowered circulating leptin and suppressed by administration of exogenous leptin [131]. Similarly, the high levels of glucocorticoids observed in* ob/ob* mice can be rescued by leptin substitution [73, 132].

**Figure 1:**
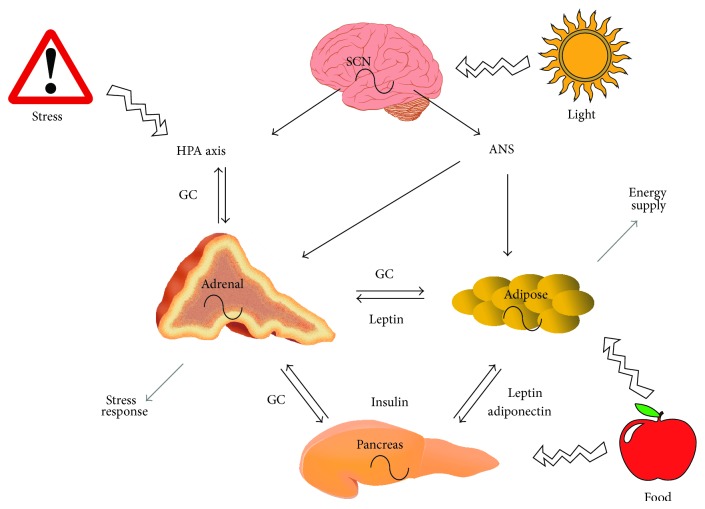
Interaction of stress axis and adipose circadian rhythms. Rhythmic adrenal glucocorticoid (GC) release negatively feeds back on the HPA axis and stimulates lipid mobilization in adipose tissue and release of insulin from the pancreas. Insulin supports lipogenesis in adipocytes, while the adipokine leptin inhibits insulin secretion from the pancreas and glucocorticoid release from the adrenal. Adiponectin increases insulin sensitivity in cells. External factors like stress and food intake affect the peripheral rhythms, while light exposure entrains the system via the SCN. For more details see text (autonomic nervous system (ANS)).

A direct adipo-adrenal feedback loop has been postulated [133–135], with leptin being the suppressive arm and the HPA axis, specifically glucocorticoids, being the positive arm. The mechanism of action for leptin suppression of glucocorticoid concentration may be located at the level of the hypothalamus, since CRH secretion from isolated hypothalamic neurons is inhibited by leptin, but this is not true for ACTH secretion from isolated pituicytes [134]. Leptin is also capable of interacting directly with the adrenal gland, with functional leptin receptors being present in the adrenal cortex and to a lesser extent in the catecholamine-producing adrenal medulla [136, 137]. Furthermore, the leptin response is absent in adrenal cells from* db/db* mice [138]. Leptin has been found to inhibit basal and ACTH stimulated secretion of glucocorticoids in rodent and human adrenal tissue [136, 138, 139], with additional effects on the secretion of aldosterone in human adrenals being reported [136] (Figure 1).

Taken together, these data suggest a regulatory role of leptin on the HPA axis, and although this interaction is unlikely to directly drive the circadian rhythm of glucocorticoids in circulation, leptin may modulate this rhythm at the level of the hypothalamus or the pituitary. This is of particular interest in the context of metabolic disease, since altered leptin profiles (such as leptin resistance, insufficient or dysfunctional leptin production or signalling) accompanying obesity may interact with the HPA axis in order to contribute to metabolic disorder.

Interactions between the HPA axis and adiponectin have also been described, although the effects appear contradictory and therefore a clear relationship is controversial. Glucocorticoids and ACTH both suppress adiponectin secretion from WAT in human and murine cell culture experiments [140, 141]. Reciprocally, adiponectin receptors have been observed throughout the rodent and human adrenal gland [142, 143]. Unlike leptin [136], adiponectin (*ADIPOQ*) mRNA has further been detected in the adrenal gland, in the* zona glomerulosa* of the adrenal cortex in rats [144], although this is in contrast to studies in mice and the murine adrenocortical Y-1 cell line [143].* In vivo* administration of excess glucocorticoids to rats reduces circulating adiponectin concentrations, and adrenalectomy leads to a reduction of adiponectin gene expression in epididymal WAT [145]. The reported effects of adiponectin on glucocorticoid secretion are also contradictory. For example,* in vitro* administration of adiponectin to adrenocortical Y-1 cells suppresses basal and ACTH stimulated glucocorticoid secretion and concordant alteration of steroidogenic gene expression, including that of* StAR* and* CYP11A1* [143]. However, in a different study, adiponectin administration to a primary culture of rat adenocytes led to dose-dependent enhancement of corticosterone secretion [144].

Several inflammatory cytokines are produced and secreted as part of the adipokine function of adipose tissue. Interleukin-6 (IL-6), tumour necrosis factor *α* (TNF-*α*), and chemerin secretion from adipose tissue fluctuate diurnally, with rhythms peaking during the rest phase, which is daytime for rodents [146–148] and during the late night or early morning for humans [149]. Glucocorticoids, in line with their immunosuppressive function, counteract on this release to minimise damage of host tissue [150, 151].

## 7. Clinical Implications

Research of the last decades has uncovered a tight interaction of the circadian rhythms of the HPA axis, adipocyte function, and adipokine secretion (Figure 1). The integration of these systems is essential in the maintenance of health, and the deregulation or alteration of one rhythm, in the context of disease or by lifestyle interventions, is likely to be reciprocated in the other systems. For example, obesity is correlated with hypercortisolemia and altered HPA axis rhythmicity and we have described the reciprocal effects of alterations in these rhythms above, thus increasing the vulnerability for HPA axis disorders and* vice versa*. Thus, stabilising circadian glucocorticoid rhythms may be an important approach to counteract metabolic disorders.

Under acute stress, glucocorticoid release feeds back to the hypothalamus to initiate the return to homeostatic conditions, and at the same time, mobilizes energy from body stores. Irregular sleep/wake cycles, jetlag, or shift work can lead to a constant disruption of circadian processes very much resembling the effects of chronic stress exposure [152]. A persistently activated HPA axis results in elevated blood glucose, hyperinsulinaemia, and insulin [153] and leptin resistance [154]. Obesity is accompanied by an adipose induced systemic inflammatory state which may contribute to the development of rheumatoid arthritis [155]. Obesity-associated dampened physiological and behavioural rhythms can further affect sleep quality, food intake, and immune function [156–158]. In rheumatoid arthritis patients, long-term administration of high doses of hydrocortisone can lead to Cushing's disease-like phenotypes [159] including diabetes mellitus, osteoporosis, hypertension, dyslipidemia, and sleep disorders [160].

To restore disrupted endocrine glucocorticoid rhythms, timed substitution or inhibition of steroid levels is required. In hypocortisolaemic disorders exogenous hydrocortisone administration is the mainstay of treatment, primarily serving to restore adequate stress responses and bypass the symptoms of chronic adrenal insufficiency such as weight loss, fatigue, and nausea [161]. On the other hand, hydrocortisone doses should be minimised to avoid adverse side effects of glucocorticoid surplus such as weight gain [162], osteoporosis [163], or impaired glucose tolerance [164]. In an attempt to mimic physiological glucocorticoid patterns the total dose (between 15 and 25 mg per day) is distributed into two or three quantities with the highest dose applied during the morning. Timing the last hydrocortisone dose at least 5-6 h before bedtime prevents sleep disruption due to supra-physiological cortisol concentrations [161]. An alternative approach is a dual-release hydrocortisone preparation, which is currently tested in patients with adrenal insufficiency [165–167]. A profiled hydrocortisone infusion controlled by a subcutaneous pump has been tested in a small cohort of Addison patients [165], and another approach used a delayed release formulation of prednisone which, when taken in the evening, leads to an increasing availability of the drug starting at approximately 2 a.m. to counteract morning stiffness in rheumatoid arthritis patients [168]. The effect of such chronotherapeutic approaches on adipose function, however, remains to be shown. Another yet unexplored route, that would rather target hypercortisolaemic conditions, as seen in Cushing's disease or stress disorder patients, involves timed suppression of glucocorticoid levels, for example, by the antisteroidogenic drug metyrapone. In mice, this has been shown to alleviate jetlag-induced disruption of the rest-activity cycle [169].

## 8. Concluding Remarks

While the circadian rhythm of glucocorticoid secretion was among the first described, adipose rhythmicity and adipose clocks have raised more recent interest because of their pivotal role in energy homeostasis. By release of endocrine factors (glucocorticoids and adipokines) both systems communicate with each other and regulate stress responses and energy metabolism. Chronotherapeutic approaches targeting this crosstalk may be fruitful in treating metabolic diseases and stress disorders. It is tempting to speculate that this may not be restricted to targets in peripheral tissues but may extend to central aspects of appetite regulation and stress- and obesity-associated neuropsychiatric alterations.

## References

[1] Panda S., Hogenesch J. B., Kay S. A. (2002). Circadian rhythms from flies to human. *Nature*.

[2] Dibner C., Schibler U., Albrecht U. (2010). The mammalian circadian timing system: organization and coordination of central and peripheral clocks. *Annual Review of Physiology*.

[3] Balsalobre A., Brown S. A., Marcacci L. (2000). Resetting of circadian time in peripheral tissues by glucocorticoid signaling. *Science*.

[4] So A. Y.-L., Bernal T. U., Pillsbury M. L., Yamamoto K. R., Feldman B. J. (2009). Glucocorticoid regulation of the circadian clock modulates glucose homeostasis. *Proceedings of the National Academy of Sciences of the United States of America*.

[5] Lamia K. A., Papp S. J., Yu R. T. (2011). Cryptochromes mediate rhythmic repression of the glucocorticoid receptor. *Nature*.

[6] Zhang E. E., Kay S. A. (2010). Clocks not winding down: unravelling circadian networks. *Nature Reviews Molecular Cell Biology*.

[7] Brown S. A., Kowalska E., Dallmann R. (2012). (Re)inventing the circadian feedback loop. *Developmental Cell*.

[8] Dimitrov S., Benedict C., Heutling D., Westermann J., Born J., Lange T. (2009). Cortisol and epinephrine control opposing circadian rhythms in T cell subsets. *Blood*.

[9] Liston C., Cichon J. M., Jeanneteau F., Jia Z., Chao M. V., Gan W.-B. (2013). Circadian glucocorticoid oscillations promote learning-dependent synapse formation and maintenance. *Nature Neuroscience*.

[10] Wagner U., Born J. (2008). Memory consolidation during sleep: interactive effects of sleep stages and HPA regulation. *Stress*.

[11] Rosmond R. (2005). Role of stress in the pathogenesis of the metabolic syndrome. *Psychoneuroendocrinology*.

[12] Simon C., Weibel L., Brandenberger G. (2000). Twenty-four-hour rhythms of plasma glucose and insulin secretion rate in regular night workers. *The American Journal of Physiology—Endocrinology and Metabolism*.

[13] Lund J., Arendt J., Hampton S. M., English J., Morgan L. M. (2001). Postprandial hormone and metabolic responses amongst shift workers in Antarctica. *Journal of Endocrinology*.

[14] Pan A., Schernhammer E. S., Sun Q., Hu F. B. (2011). Rotating night shift work and risk of type 2 diabetes: two prospective cohort studies in women. *PLoS Medicine*.

[15] Walker B. R., Soderberg S., Lindahl B., Olsson T. (2000). Independent effects of obesity and cortisol in predicting cardiovascular risk factors in men and women. *Journal of Internal Medicine*.

[16] Ljung T., Holm G., Friberg P. (2000). The activity of the hypothalamic-pituitary-adrenal axis and the sympathetic nervous system in relation to waist/hip circumference ratio in men. *Obesity Research*.

[17] Rosmond R., Dallman M. F., Björntorp P. (1998). Stress-related cortisol secretion in men: relationships with abdominal obesity and endocrine, metabolic and hemodynamic abnormalities. *Journal of Clinical Endocrinology and Metabolism*.

[18] Pasquali R., Vicennati V., Cacciari M., Pagotto U. (2006). The hypothalamic-pituitary-adrenal axis activity in obesity and the metabolic syndrome. *Annals of the New York Academy of Sciences*.

[19] Buijs R. M., Kalsbeek A., van der Woude T. P., van Heerikhuize J. J., Shinn S. (1993). Suprachiasmatic nucleus lesion increases corticosterone secretion. *The American Journal of Physiology*.

[20] Ulrich-Lai Y. M., Herman J. P. (2009). Neural regulation of endocrine and autonomic stress responses. *Nature Reviews Neuroscience*.

[21] Dickmeis T., Weger B. D., Weger M. (2013). The circadian clock and glucocorticoids—interactions across many time scales. *Molecular and Cellular Endocrinology*.

[22] Boivin D. B., Czeisler C. A. (1998). Resetting of circadian melatonin and cortisol rhythms in humans by ordinary room light. *NeuroReport*.

[23] Boivin D. B. (2000). Influence of sleep-wake and circadian rhythm disturbances in psychiatric disorders. *Journal of Psychiatry and Neuroscience*.

[24] Klerman E. B., Gershengorn H. B., Duffy J. F., Kronauer R. E. (2002). Comparisons of the variability of three markers of the human circadian pacemaker. *Journal of Biological Rhythms*.

[25] Lightman S. L., Conway-Campbell B. L. (2010). The crucial role of pulsatile activity of the HPA axis for continuous dynamic equilibration. *Nature Reviews Neuroscience*.

[26] Stephan F. K., Zucker I. (1972). Circadian rhythms in drinking behavior and locomotor activity of rats are eliminated by hypothalamic lesions. *Proceedings of the National Academy of Sciences of the United States of America*.

[27] Moore R. Y., Eichler V. B. (1972). Loss of a circadian adrenal corticosterone rhythm following suprachiasmatic lesions in the rat. *Brain Research*.

[28] Waite E. J., Mckenna M., Kershaw Y. (2012). Ultradian corticosterone secretion is maintained in the absence of circadian cues. *European Journal of Neuroscience*.

[29] Engler D., Pham T., Liu J.-P., Fullerton M. J., Clarke I. J., Funder J. W. (1990). Studies of the regulation of the hypothalamic-pituitary-adrenal axis in sheep with hypothalamic-pituitary disconnection. II. Evidence for in vivo ultradian hypersecretion of proopiomelanocortin peptides by the isolated anterior and intermediate pituitary. *Endocrinology*.

[30] Walker J. J., Spiga F., Waite E. (2012). The origin of glucocorticoid hormone oscillations. *PLoS Biology*.

[31] De Kloet E. R., Joëls M., Holsboer F. (2005). Stress and the brain: from adaptation to disease. *Nature Reviews Neuroscience*.

[32] Reul J. M. H. M., de Kloet E. R. (1985). Two receptor systems for corticosterone in rat brain: microdistribution and differential occupation. *Endocrinology*.

[33] Caprio M., Fève B., Claës A., Viengchareun S., Lombès M., Zennaro M.-C. (2007). Pivotal role of the mineralocorticoid receptor in corticosteroid-induced adipogenesis. *The FASEB Journal*.

[34] Doi M., Takahashi Y., Komatsu R. (2010). Salt-sensitive hypertension in circadian clock-deficient Cry-null mice involves dysregulated adrenal Hsd3b6. *Nature Medicine*.

[35] Wolfe L. K., Gordon R. D., Island D. P., Liddle G. W. (1966). An analysis of factors determining the circadian pattern of aldosterone excretion.. *Journal of Clinical Endocrinology and Metabolism*.

[36] Curtis A. M., Cheng Y., Kapoor S., Reilly D., Price T. S., FitzGerald G. A. (2007). Circadian variation of blood pressure and the vascular response to asynchronous stress. *Proceedings of the National Academy of Sciences of the United States of America*.

[37] Henley D. E., Leendertz J. A., Russell G. M. (2009). Development of an automated blood sampling system for use in humans. *Journal of Medical Engineering and Technology*.

[38] Watts A. G., Tanimura S., Sanchez-Watts G. (2004). Corticotropin-releasing hormone and arginine vasopressin gene transcription in the hypothalamic paraventricular nucleus of unstressed rats: daily rhythms and their interactions with corticosterone. *Endocrinology*.

[39] Girotti M., Weinberg M. S., Spencer R. L. (2009). Diurnal expression of functional and clock-related genes throughout the rat HPA axis: system-wide shifts in response to a restricted feeding schedule. *The American Journal of Physiology—Endocrinology and Metabolism*.

[40] Lilley T. R., Wotus C., Taylor D., Lee J. M., De La Iglesia H. O. (2012). Circadian regulation of cortisol release in behaviorally split golden hamsters. *Endocrinology*.

[41] Muglia L. J., Jacobson L., Weninger S. C. (1997). Impaired diurnal adrenal rhythmicity restored by constant infusion of corticotropin-releasing hormone in corticotropin-releasing hormone-deficient mice. *The Journal of Clinical Investigation*.

[42] Yoder J. M., Brandeland M., Engeland W. C. (2014). Phase-dependent resetting of the adrenal clock by ACTH in vitro. *The American Journal of Physiology—Regulatory Integrative and Comparative Physiology*.

[43] Campino C., Valenzuela F. J., Torres-Farfan C. (2011). Melatonin exerts direct inhibitory actions on ACTH responses in the human adrenal gland. *Hormone and Metabolic Research*.

[44] Buijs R. M., Wortel J., van Heerikhuize J. J. (1999). Anatomical and functional demonstration of a multisynaptic suprachiasmatic nucleus adrenal (cortex) pathway. *European Journal of Neuroscience*.

[45] Meier A. H. (1976). Daily variation in concentration of plasma corticosteroid in hypophysectomized rats. *Endocrinology*.

[46] Ottenweller J. E., Meier A. H. (1982). Adrenal innervation may be an extrapituitary mechanism able to regulate adrenocortical rhythmicity in rats. *Endocrinology*.

[47] Wotus C., Lilley T. R., Neal A. S. (2013). Forced desynchrony reveals independent contributions of suprachiasmatic oscillators to the daily plasma corticosterone rhythm in male rats. *PLoS ONE*.

[48] Ulrich-Lai Y. M., Arnhold M. M., Engeland W. C. (2006). Adrenal splanchnic innervation contributes to the diurnal rhythm of plasma corticosterone in rats by modulating adrenal sensitivity to ACTH. *The American Journal of Physiology—Regulatory Integrative and Comparative Physiology*.

[49] Engeland W. C. (2013). Sensitization of endocrine organs to anterior pituitary hormones by the autonomic nervous system. *Handbook of Clinical Neurology*.

[50] Andrews R. V., Edgar Folk G. (1964). Circadian metabolic patterns in cultured hamster adrenal glands. *Comparative Biochemistry and Physiology*.

[51] Bittman E. L., Doherty L., Huang L., Paroskie A. (2003). *Period* gene expression in mouse endocrine tissues. *American Journal of Physiology: Regulatory Integrative and Comparative Physiology*.

[52] Lemos D. R., Downs J. L., Urbanski H. F. (2006). Twenty-four-hour rhythmic gene expression in the rhesus macaque adrenal gland. *Molecular Endocrinology*.

[53] Oster H., Abraham D., Leitges M. (2006). Expression of the protein kinase D (PKD) family during mouse embryogenesis. *Gene Expression Patterns*.

[54] Oster H., Damerow S., Hut R. A., Eichele G. (2006). Transcriptional profiling in the adrenal gland reveals circadian regulation of hormone biosynthesis genes and nucleosome assembly genes. *Journal of Biological Rhythms*.

[55] Fahrenkrug J., Hannibal J., Georg B. (2008). Diurnal rhythmicity of the canonical clock genes Per1, Per2 and Bmal1 in the rat adrenal gland is unaltered after hypophysectomy. *Journal of Neuroendocrinology*.

[56] Valenzuela F. J., Torres-Farfan C., Richter H. G. (2008). Clock gene expression in adult primate suprachiasmatic nuclei and adrenal: is the adrenal a peripheral clock responsive to melatonin?. *Endocrinology*.

[57] Barclay J. L., Shostak A., Leliavski A. (2013). High-fat diet-induced hyperinsulinemia and tissue-specific insulin resistance in Cry-deficient mice. *The American Journal of Physiology—Endocrinology and Metabolism*.

[58] Leliavski A., Shostak A., Husse J., Oster H. (2014). Impaired glucocorticoid production and response to stress in Arntl-deficient male mice. *Endocrinology*.

[59] Turek F. W., Joshu C., Kohsaka A. (2005). Obesity and metabolic syndrome in circadian *Clock* mutant mice. *Science*.

[60] Son G. H., Chung S., Choe H. K. (2008). Adrenal peripheral clock controls the autonomous circadian rhythm of glucocorticoid by causing rhythmic steroid production. *Proceedings of the National Academy of Sciences of the United States of America*.

[61] Oster H., Damerow S., Kiessling S. (2006). The circadian rhythm of glucocorticoids is regulated by a gating mechanism residing in the adrenal cortical clock. *Cell Metabolism*.

[62] Torres-Farfan C., Abarzua-Catalan L., Valenzuela F. J. (2009). Cryptochrome 2 expression level is critical for adrenocorticotropin stimulation of cortisol production in the capuchin monkey adrenal. *Endocrinology*.

[63] Stratmann M., Schibler U. (2006). Properties, entrainment, and physiological functions of mammalian peripheral oscillators. *Journal of Biological Rhythms*.

[64] Nader N., Chrousos G. P., Kino T. (2010). Interactions of the circadian CLOCK system and the HPA axis. *Trends in Endocrinology and Metabolism*.

[65] Ratman D., Vanden Berghe W., Dejager L. (2013). How glucocorticoid receptors modulate the activity of other transcription factors: a scope beyond tethering. *Molecular and Cellular Endocrinology*.

[66] Yamamoto T., Nakahata Y., Tanaka M. (2005). Acute physical stress elevates mouse *Period1* mRNA expression in mouse peripheral tissues via a glucocorticoid-responsive element. *The Journal of Biological Chemistry*.

[67] Segall L. A., Milet A., Tronche F., Amir S. (2009). Brain glucocorticoid receptors are necessary for the rhythmic expression of the clock protein, PERIOD2, in the central extended amygdala in mice. *Neuroscience Letters*.

[68] Gómez-Abellán P., Díez-Noguera A., Madrid J. A., Luján J. A., Ordovás J. M., Garaulet M. (2012). Glucocorticoids affect 24 h clock genes expression in human adipose tissue explant cultures. *PLoS ONE*.

[69] Nader N., Chrousos G. P., Kino T. (2009). Circadian rhythm transcription factor CLOCK regulates the transcriptional activity of the glucocorticoid receptor by acetylating its hinge region lysine cluster: potential physiological implications. *The FASEB Journal*.

[70] Damiola F., Le Minli N., Preitner N., Kornmann B., Fleury-Olela F., Schibler U. (2000). Restricted feeding uncouples circadian oscillators in peripheral tissues from the central pacemaker in the suprachiasmatic nucleus. *Genes and Development*.

[71] Le Minh N., Damiola F., Tronche F., Schütz G., Schibler U. (2002). Glucocorticoid hormones inhibit food-induced phase-shifting of peripheral circadian oscillators. *EMBO Journal*.

[72] Kohsaka A., Laposky A. D., Ramsey K. M. (2007). High-fat diet disrupts behavioral and molecular circadian rhythms in mice. *Cell Metabolism*.

[73] Ahima R. S., Prabakaran D., Flier J. S. (1998). Postnatal leptin surge and regulation of circadian rhythm of leptin by feeding. Implications for energy homeostasis and neuroendocrine function. *Journal of Clinical Investigation*.

[74] Walker C.-D., Scribner K. A., Stern J. S., Dallman M. F. (1992). Obese Zucker fa/fa rats exhibit normal target sensitivity to corticosterone and increased drive to adrenocorticotropin during the diurnal trough. *Endocrinology*.

[75] Martin R. J., Wangsness P. J., Gahagan J. H. (1978). Diurnal changes in serum metabolites and hormones in lean and obese Zucker rats. *Hormone and Metabolic Research*.

[76] García-Prieto M. D., Tébar F. J., Nicolás F., Larqué E., Zamora S., Garaulet M. (2007). Cortisol secretary pattern and glucocorticoid feedback sensitivity in women from a Mediterranean area: relationship with anthropometric characteristics, dietary intake and plasma fatty acid profile. *Clinical Endocrinology*.

[77] Dudley C. A., Erbel-Sieler C., Estill S. J. (2003). Altered patterns of sleep and behavioral adaptability in NPAS2-deficient mice. *Science*.

[78] Dallmann R., Touma C., Palme R., Albrecht U., Steinlechner S. (2006). Impaired daily glucocorticoid rhythm in *Per1*
^*Brd*^ mice. *Journal of Comparative Physiology A: Neuroethology, Sensory, Neural, and Behavioral Physiology*.

[79] Griebel G., Ravinet-Trillou C., Beeské S., Avenet P., Pichat P. (2014). Mice deficient in cryptochrome 1 (Cry1^−/−^) exhibit resistance to obesity induced by a high-fat diet. *Frontiers in Endocrinology*.

[80] Cook A., Cowan C. (2008). Adipose. *StemBook*.

[81] Proença A. R. G., Sertié R. A. L., Oliveira A. C. (2014). New concepts in white adipose tissue physiology. *Brazilian Journal of Medical and Biological Research*.

[82] Divertie G. D., Jensen M. D., Miles J. M. (1991). Stimulation of lipolysis in humans by physiological hypercortisolemia. *Diabetes*.

[83] Shostak A., Husse J., Oster H. (2014). Circadian regulation of adipose function. *Adipocyte*.

[84] Dallmann R., Viola A. U., Tarokh L., Cajochen C., Brown S. A. (2012). The human circadian metabolome. *Proceedings of the National Academy of Sciences of the United States of America*.

[85] Rey G., Cesbron F., Rougemont J., Reinke H., Brunner M., Naef F. (2011). Genome-wide and phase-specific DNA-binding rhythms of BMAL1 control circadian output functions in mouse liver. *PLoS Biology*.

[86] Zvonic S., Ptitsyn A. A., Conrad S. A. (2006). Characterization of peripheral circadian clocks in adipose tissues. *Diabetes*.

[87] Grimaldi B., Bellet M. M., Katada S. (2010). PER2 controls lipid metabolism by direct regulation of PPAR*γ*. *Cell Metabolism*.

[88] van der Spek R., Kreier F., Fliers E., Kalsbeek A. (2012). Circadian rhythms in white adipose tissue. *Progress in Brain Research*.

[89] Havel P. J. (2002). Control of energy homeostasis and insulin action by adipocyte hormones: leptin, acylation stimulating protein, and adiponectin. *Current Opinion in Lipidology*.

[90] Lee D. K., Jeong J. H., Chun S. K., Chua S., Jo Y. H. (2015). Interplay between glucose and leptin signalling determines the strength of GABAergic synapses at POMC neurons. *Nature Communications*.

[91] Tsang A. H., Kolbe I., Seemann J., Oster H. (2014). Interaction of circadian and stress systems in the regulation of adipose physiology. *Hormone Molecular Biology and Clinical Investigation*.

[92] Ramachandran R., Gravenstein K. S., Metter E. J., Egan J. M., Ferrucci L., Chia C. W. (2012). Selective contribution of regional adiposity, skeletal muscle, and adipokines to glucose disposal in older adults. *Journal of the American Geriatrics Society*.

[93] Minokoshi Y., Alquier T., Furukawa H. (2004). AMP-kinase regulates food intake by responding to hormonal and nutrient signals in the hypothalamus. *Nature*.

[94] Considine R. V., Sinha M. K., Heiman M. L. (1996). Serum immunoreactive-leptin concentrations in normal-weight and obese humans. *The New England Journal of Medicine*.

[95] Schoeller D. A., Cella L. K., Sinha M. K., Caro J. F. (1997). Entrainment of the diurnal rhythm of plasma leptin to meal timing. *The Journal of Clinical Investigation*.

[96] Birketvedt G. S., Sundsfjord J., Florholmen J. R. (2002). Hypothalamic-pituitary-adrenal axis in the night eating syndrome. *American Journal of Physiology—Endocrinology & Metabolism*.

[97] Boston R. C., Moate P. J., Allison K. C., Lundgren J. D., Stunkard A. J. (2008). Modeling circadian rhythms of food intake by means of parametric deconvolution: results from studies of the night eating syndrome. *The American Journal of Clinical Nutrition*.

[98] Yildiz B. O., Suchard M. A., Wong M.-L., McCann S. M., Licinio J. (2004). Alterations in the dynamics of circulating ghrelin, adiponectin, and leptin in human obesity. *Proceedings of the National Academy of Sciences of the United States of America*.

[99] Chu M. C., Cosper P., Orio F., Carmina E., Lobo R. A. (2006). Insulin resistance in postmenopausal women with metabolic syndrome and the measurements of adiponectin, leptin, resistin, and ghrelin. *American Journal of Obstetrics and Gynecology*.

[100] Silha J. V., Krsek M., Skrha J. V., Sucharda P., Nyomba B. L. G., Murphy L. J. (2003). Plasma resistin, adiponectin and leptin levels in lean and obese subjects: correlations with insulin resistance. *European Journal of Endocrinology*.

[101] Sukumaran S., Xue B., Jusko W. J., Dubois D. C., Almon R. R. (2010). Circadian variations in gene expression in rat abdominal adipose tissue and relationship to physiology. *Physiological Genomics*.

[102] Chung S., Son G. H., Kim K. (2011). Circadian rhythm of adrenal glucocorticoid: its regulation and clinical implications. *Biochimica et Biophysica Acta—Molecular Basis of Disease*.

[103] Rask E., Olsson T., Söderberg S. (2001). Tissue-specific dysregulation of cortisol metabolism in human obesity. *Journal of Clinical Endocrinology and Metabolism*.

[104] Burén J., Bergström S.-A., Loh E., Söderström I., Olsson T., Mattsson C. (2007). Hippocampal 11beta-hydroxysteroid dehydrogenase type 1 messenger ribonucleic acid expression has a diurnal variability that is lost in the obese Zucker rat. *Endocrinology*.

[105] Harris H. J., Kotelevtsev Y., Mullins J. J., Seckl J. R., Holmes M. C. (2001). Intracellular regeneration of glucocorticoids by 11*β*-hydroxysteroid dehydrogenase (11*β*-HSD)-1 plays a key role in regulation of the hypothalamic-pituitary-adrenal axis: analysis of 11*β*-HSD-1-deficient mice. *Endocrinology*.

[106] de Guia R. M., Rose A. J., Herzig S. (2014). Glucocorticoid hormones and energy homeostasis. *Hormone Molecular Biology and Clinical Investigation*.

[107] Zhang P., O'Loughlin L., Brindley D. N., Reue K. (2008). Regulation of lipin-1 gene expression by glucocorticoids during adipogenesis. *The Journal of Lipid Research*.

[108] Asada M., Rauch A., Shimizu H. (2011). DNA binding-dependent glucocorticoid receptor activity promotes adipogenesis via Krüppel-like factor 15 gene expression. *Laboratory Investigation*.

[109] Bornstein S. R., Schuppenies A., Wong M.-L., Licinio J. (2006). Approaching the shared biology of obesity and depression: the stress axis as the locus of gene-environment interactions. *Molecular Psychiatry*.

[110] Branth S., Ronquist G., Stridsberg M. (2007). Development of abdominal fat and incipient metabolic syndrome in young healthy men exposed to long-term stress. *Nutrition, Metabolism and Cardiovascular Diseases*.

[111] Lucassen E. A., Cizza G. (2012). The hypothalamic-pituitary-adrenal axis, obesity, and chronic stress exposure: sleep and the hpa axis in obesity. *Current Obesity Reports*.

[112] Murakami T., Iida M., Shima K. (1995). Dexamethasone regulates obese expression in isolated rat adipocytes. *Biochemical and Biophysical Research Communications*.

[113] Slieker L. J., Sloop K. W., Surface P. L. (1996). Regulation of expression of ob mRNA and protein by glucocorticoids and cAMP. *The Journal of Biological Chemistry*.

[114] Wabitsch M., Jensen P. B., Blum W. F. (1996). Insulin and cortisol promote leptin production in cultured human fat cells. *Diabetes*.

[115] Hardie L. J., Guilhot N., Trayhurn P. (1996). Regulation of leptin production in cultured mature white adipocytes. *Hormone and Metabolic Research*.

[116] De Vos P., Saladin R., Auwerx J., Staels B. (1995). Induction of ob gene expression by corticosteroids is accompanied by body weight loss and reduced food intake. *The Journal of Biological Chemistry*.

[117] Zakrzewska K. E., Cusin I., Stricker-Krongrad A. (1999). Induction of obesity and hyperleptinemia by central glucocorticoid infusion in the rat. *Diabetes*.

[118] Larsson H., Ahrén B. (1996). Short-term dexamethasone treatment increases plasma leptin independently of changes in insulin sensitivity in healthy women. *Journal of Clinical Endocrinology and Metabolism*.

[119] Berneis K., Vosmeer S., Keller U. (1996). Effects of glucocorticoids and of growth hormone on serum leptin concentrations in man. *European Journal of Endocrinology*.

[120] Miell J. P., Englaro P., Blum W. F. (1996). Dexamethasone induces an acute and sustained rise in circulating leptin levels in normal human subjects. *Hormone and Metabolic Research*.

[121] Kiess W., Englaro P., Hanitsch S., Rascher W., Attanasio A., Blum W. F. (1996). High leptin concentrations in serum of very obese children are further stimulated by dexamethasone. *Hormone and Metabolic Research*.

[122] Papaspyrou-Rao S., Schneider S. H., Petersen R. N., Fried S. K. (1997). Dexamethasone increases leptin expression in humans in vivo. *Journal of Clinical Endocrinology and Metabolism*.

[123] Dagogo-Jack S., Selke G., Melson A. K., Newcomer J. W. (1997). Robust leptin secretory responses to dexamethasone in obese subjects. *Journal of Clinical Endocrinology and Metabolism*.

[124] Kolaczynski J. W., Goldstein B. J., Considine R. V. (1997). Dexamethasone, OB gene, and leptin in humans; Effect of exogenous hyperinsulinemia. *Journal of Clinical Endocrinology and Metabolism*.

[125] Friedman J. M., Halaas J. L. (1998). Leptin and the regulation of body weight in mammals. *Nature*.

[126] Masuzaki H., Ogawa Y., Hosoda K. (1997). Glucocorticoid regulation of leptin synthesis and secretion in humans: elevated plasma leptin levels in Cushing's syndrome. *The Journal of Clinical Endocrinology and Metabolism*.

[127] Widjaja A., Schürmeyer T. H., Von Zur Mühlen A., Brabant G. (1998). Determinants of serum leptin levels in Cushing's syndrome. *Journal of Clinical Endocrinology and Metabolism*.

[128] Zakrzewska K. E., Cusin I., Sainsbury A., Rohner-Jeanrenaud F., Jeanrenaud B. (1997). Glucocorticoids as counterregulatory hormones of leptin: toward an understanding of leptin resistance. *Diabetes*.

[129] Lee M.-J., Pramyothin P., Karastergiou K., Fried S. K. (2014). Deconstructing the roles of glucocorticoids in adipose tissue biology and the development of central obesity. *Biochimica et Biophysica Acta—Molecular Basis of Disease*.

[130] Saito M., Bray G. A. (1983). Diurnal rhythm for corticosterone in obese (ob/ob) diabetes (db/db) and gold-thioglucose-induced obesity in mice. *Endocrinology*.

[131] Ahlma R. S., Prabakaran D., Mantzoros C. (1996). Role of leptin in the neuroendocrine response to fasting. *Nature*.

[132] Stephens T. W., Basinski M., Bristow P. K. (1995). The role of neuropeptide Y in the antiobesity action of the obese gene product. *Nature*.

[133] Wauters M., Considine R. V., van Gaal L. F. (2000). Human leptin: from an adipocyte hormone to an endocrine mediator. *European Journal of Endocrinology*.

[134] Heiman M. L., Ahima R. S., Craft L. S., Schoner B., Stephens T. W., Flier J. S. (1997). Leptin inhibition of the hypothalamic-pituitary-adrenal axis in response to stress. *Endocrinology*.

[135] Kargi A. Y., Iacobellis G. (2014). Adipose tissue and adrenal glands: Novel pathophysiological mechanisms and clinical applications. *International Journal of Endocrinology*.

[136] Glasow A., Haidan A., Hilbers U. (1998). Expression of Ob receptor in normal human adrenals: differential regulation of adrenocortical and adrenomedullary function by leptin. *Journal of Clinical Endocrinology and Metabolism*.

[137] Takekoshi K., Motooka M., Isobe K. (1999). Leptin directly stimulates catecholamine secretion and synthesis in cultured porcine adrenal medullary chromaffin cells. *Biochemical and Biophysical Research Communications*.

[138] Pralong F. P., Roduit R., Waeber G. (1998). Leptin inhibits directly glucocorticoid secretion by normal human and rat adrenal gland. *Endocrinology*.

[139] Bornstein S. R., Uhlmann K., Haidan A., Ehrhart-Bornstein M., Scherbaum W. A. (1997). Evidence for a novel peripheral action of leptin as a metabolic signal to the adrenal gland: leptin inhibits cortisol release directly. *Diabetes*.

[140] Degawa-Yamauchi M., Moss K. A., Bovenkerk J. E. (2005). Regulation of adiponectin expression in human adipocytes: effects of adiposity, glucocorticoids, and tumor necrosis factor *α*. *Obesity Research*.

[141] Iwen K. A. H., Senyaman O., Schwartz A. (2008). Melanocortin crosstalk with adipose functions: ACTH directly induces insulin resistance, promotes a pro-inflammatory adipokine profile and stimulates UCP-1 in adipocytes. *Journal of Endocrinology*.

[142] Rossi G. P., Sticchi D., Giuliani L. (2006). Adiponectin receptor expression in the human adrenal cortex and aldosterone-producing adenomas. *International Journal of Molecular Medicine*.

[143] Li P., Sun F., Cao H.-M. (2009). Expression of adiponectin receptors in mouse adrenal glands and the adrenocortical Y-1 cell line: adiponectin regulates steroidogenesis. *Biochemical and Biophysical Research Communications*.

[144] Paschke L., Zemleduch T., Rucinski M., Ziolkowska A., Szyszka M., Malendowicz L. K. (2010). Adiponectin and adiponectin receptor system in the rat adrenal gland: ontogenetic and physiologic regulation, and its involvement in regulating adrenocortical growth and steroidogenesis. *Peptides*.

[145] De Oliveira C., Iwanaga-Carvalho C., Mota J. F., Oyama L. M., Ribeiro E. B., Oller Do Nascimento C. M. (2011). Effects of adrenal hormones on the expression of adiponectin and adiponectin receptors in adipose tissue, muscle and liver. *Steroids*.

[146] Guan Z., Vgontzas A. N., Omori T., Peng X., Bixler E. O., Fang J. (2005). Interleukin-6 levels fluctuate with the light-dark cycle in the brain and peripheral tissues in rats. *Brain, Behavior, and Immunity*.

[147] Cano P., Cardinali D. P., Ríos-Lugo M. J., Fernández-Mateos M. P., Reyes Toso C. F., Esquifino A. I. (2009). Effect of a high-fat diet on 24-hour pattern of circulating adipocytokines in rats. *Obesity*.

[148] Parlee S. D., Ernst M. C., Muruganandan S., Sinal C. J., Goralski K. B. (2010). Serum chemerin levels vary with time of day and are modified by obesity and tumor necrosis factor-*α*. *Endocrinology*.

[149] Vgontzas A. N., Bixler E. O., Lin H.-M., Prolo P., Trakada G., Chrousos G. P. (2005). IL-6 and its circadian secretion in humans. *NeuroImmunoModulation*.

[150] Hoppmann J., Perwitz N., Meier B. (2010). The balance between gluco- and mineralo-corticoid action critically determines inflammatory adipocyte responses. *Journal of Endocrinology*.

[151] Cao H. (2014). Adipocytokines in obesity and metabolic disease. *Journal of Endocrinology*.

[152] Albrecht U. (2013). Circadian clocks and mood-related behaviors. *Circadian Clocks*.

[153] Hryhorczuk C., Sharma S., Fulton S. E. (2013). Metabolic disturbances connecting obesity and depression. *Frontiers in Neuroscience*.

[154] Wauters M., Considine R. V., van Gaal L. F. (2000). Human leptin: from an adipocyte hormone to an endocrine mediator. *European Journal of Endocrinology*.

[155] Gremese E., Tolusso B., Gigante M. R., Ferraccioli G. (2014). Obesity as a risk and severity factor in rheumatic diseases (autoimmune chronic inflammatory diseases). *Frontiers in Immunology*.

[156] Wick G., Hu Y., Schwarz S., Kroemer G. (1993). Immunoendocrine communication via the hypothalamo-pituitary-adrenal axis in autoimmune diseases. *Endocrine Reviews*.

[157] Xu H., Barnes G. T., Yang Q. (2003). Chronic inflammation in fat plays a crucial role in the development of obesity-related insulin resistance. *Journal of Clinical Investigation*.

[158] Trayhurn P. (2005). Adipose tissue in obesity—an inflammatory issue. *Endocrinology*.

[159] Silverman M. N., Sternberg E. M. (2010). Matching therapy to body rhythms: an endocrine approach to treating rheumatoid arthritis. *The Journal of Rheumatology*.

[160] London E., Lodish M., Keil M. (2014). Not all glucocorticoid-induced obesity is the same: differences in adiposity among various diagnostic groups of Cushing syndrome. *Hormone and Metabolic Research*.

[161] Husebye E. S., Allolio B., Arlt W. (2014). Consensus statement on the diagnosis, treatment and follow-up of patients with primary adrenal insufficiency. *Journal of Internal Medicine*.

[162] Huscher D., Thiele K., Gromnica-Ihle E. (2009). Dose-related patterns of glucocorticoid-induced side effects. *Annals of the Rheumatic Diseases*.

[163] Løvås K., Gjesdal C. G., Christensen M. (2009). Glucocorticoid replacement therapy and pharmacogenetics in Addison's disease: effects on bone. *European Journal of Endocrinology*.

[164] Kwon S., Hermayer K. L. (2013). Glucocorticoid-induced hyperglycemia. *The American Journal of the Medical Sciences*.

[165] Løvås K., Husebye E. S. (2007). Continuous subcutaneous hydrocortisone infusion in Addison's disease. *European Journal of Endocrinology*.

[166] Johannsson G., Bergthorsdottir R., Nilsson A. G., Lennernas H., Hedner T., Skrtic S. (2009). Improving glucocorticoid replacement therapy using a novel modified-release hydrocortisone tablet: a pharmacokinetic study. *European Journal of Endocrinology*.

[167] Johannsson G., Nilsson A. G., Bergthorsdottir R. (2012). Improved cortisol exposure-time profile and outcome in patients with adrenal insufficiency: a prospective randomized trial of a novel hydrocortisone dual-release formulation. *The Journal of Clinical Endocrinology & Metabolism*.

[168] Buttgereit F., Doering G., Schaeffler A. (2008). Efficacy of modified-release versus standard prednisone to reduce duration of morning stiffness of the joints in rheumatoid arthritis (CAPRA-1): a double-blind, randomised controlled trial. *The Lancet*.

[169] Kiessling S., Eichele G., Oster H. (2010). Adrenal glucocorticoids have a key role in circadian resynchronization in a mouse model of jet lag. *The Journal of Clinical Investigation*.

